# The Influence of Dietary Protein Intake on Mammalian Tryptophan and Phenolic Metabolites

**DOI:** 10.1371/journal.pone.0140820

**Published:** 2015-10-15

**Authors:** Ruben Poesen, Henricus A. M. Mutsaers, Karen Windey, Petra H. van den Broek, Vivienne Verweij, Patrick Augustijns, Dirk Kuypers, Jitske Jansen, Pieter Evenepoel, Kristin Verbeke, Björn Meijers, Rosalinde Masereeuw

**Affiliations:** 1 Department of Microbiology and Immunology, Division of Nephrology, University Hospitals Leuven, Leuven, Belgium; 2 Department of Pharmacology and Toxicology, Radboud University Medical Center, Nijmegen, The Netherlands; 3 Department of Physiology and Pediatrics, Radboud University Medical Center, Nijmegen, The Netherlands; 4 Department of Pharmaceutical Technology and Biopharmacy, University of Groningen, Groningen, The Netherlands; 5 Translational Research for Gastrointestinal Disorders (Targid) and Leuven Food Science and Nutrition Research Centre (LFoRCe), University of Leuven, Leuven, Belgium; 6 Department of Pharmaceutical and Pharmacological Sciences, Drug Delivery and Disposition, University of Leuven, Leuven, Belgium; 7 Division of Pharmacology, Utrecht Institute for Pharmaceutical Sciences, Utrecht, The Netherlands; Hospital Universitario de La Princesa, SPAIN

## Abstract

Although there has been increasing interest in the use of high protein diets, little is known about dietary protein related changes in the mammalian metabolome. We investigated the influence of protein intake on selected tryptophan and phenolic compounds, derived from both endogenous and colonic microbial metabolism. Furthermore, potential inter-species metabolic differences were studied. For this purpose, 29 healthy subjects were allocated to a high (n = 14) or low protein diet (n = 15) for 2 weeks. In addition, 20 wild-type FVB mice were randomized to a high protein or control diet for 21 days. Plasma and urine samples were analyzed with liquid chromatography–mass spectrometry for measurement of tryptophan and phenolic metabolites. In human subjects, we observed significant changes in plasma level and urinary excretion of indoxyl sulfate (*P* 0.004 and *P* 0.001), and in urinary excretion of indoxyl glucuronide (*P* 0.01), kynurenic acid (*P* 0.006) and quinolinic acid (*P* 0.02). In mice, significant differences were noted in plasma tryptophan (*P* 0.03), indole-3-acetic acid (*P* 0.02), *p*-cresyl glucuronide (*P* 0.03), phenyl sulfate (*P* 0.004) and phenylacetic acid (*P* 0.01). Thus, dietary protein intake affects plasma levels and generation of various mammalian metabolites, suggesting an influence on both endogenous and colonic microbial metabolism. Metabolite changes are dissimilar between human subjects and mice, pointing to inter-species metabolic differences with respect to protein intake.

## Introduction

In an ongoing quest for the optimal diet and when taken into account the epidemic of obesity, there has been an increasing interest in diets low in carbohydrate with a concomitant high intake of protein. Although effective in inducing weight loss [1], there remains controversy whether these high protein diets are beneficial or may even cause harm to human health on the long term [2, 3]. In the nephrology community, there has also been a continuous debate about the ideal target of dietary protein intake in patients with chronic kidney disease (CKD). Mechanistic studies have demonstrated that high protein diets are associated with glomerular hyperfiltration, which could further compromise renal function of patients with pre-existing renal disease [4, 5]. Although criticized [6], meta-analyses of interventional studies also concluded that a low protein diet attenuates progression of CKD [7–9].

In addition, it can be hypothesized that dietary protein intake affects various health conditions by influencing the mammalian metabolome. For example, the tryptophan—kynurenine pathway has been related with inflammation and cardiovascular disease, both in the general population and patients with CKD, possibly due to activation of the aryl hydrocarbon receptor [10–12]. Recently, it has also been demonstrated that tryptophan metabolites may predict new-onset renal disease [13]. Furthermore, there has been mounting evidence that microbial metabolites originating from colonic protein fermentation play a pivotal role in the pathogenesis of adverse outcomes in patients with CKD. Both indoxyl sulfate, a tryptophan metabolite, and *p*-cresyl sulfate, a phenolic compound, have repeatedly been associated with overall mortality and cardiovascular disease [14–19], as well as with progression of CKD [20], giving rise to the so-called protein metabolite theory [21].

Of note, the impact of dietary protein intake on these and other relevant metabolites has not been fully elucidated. Moreover, it must be acknowledged that changes in protein intake will only influence the colonic fermentation process when associated with concomitant changes in colonic protein availability [22], thus largely depending on small intestinal assimilation capacity. Therefore, we aimed to explore the influence of dietary protein intake on the mammalian metabolome, focusing on selected tryptophan and phenolic compounds derived from both endogenous and colonic microbial metabolism. For this purpose, we exposed healthy human subjects and mice to either a high protein or control diet. This also allowed us to examine potential inter-species metabolic differences with respect to protein intake.

## Material and Methods

### Clinical study: population

This is a secondary analysis of a previous randomized controlled trial exploring the influence of dietary protein intake on fecal water toxicity (clinicaltrials.gov NCT01280513) [23]. After a pilot study in 9 healthy subjects, the original study included 20 subjects. For this analysis, we used the combined dataset, giving a total of 29 subjects. Participants were recruited by advertisement among students and employees of the University of Leuven. Exclusion criteria included history of abdominal surgery (with the exception of appendectomy), presence of gastro-intestinal disease (i.e., inflammatory bowel disease, irritable bowel disease or celiac disease), or presence of renal disease (i.e., estimated glomerular filtration rate (CKD-EPI) below 60 mL/min/1.73m^2^). Drug therapy influencing colonic transit or microbiota (i.e., pre-, pro- or antibiotics) was prohibited 1 month before and during study period. The study was performed according to the Declaration of Helsinki and approved by the ethics committee of the University Hospitals Leuven. Written informed consent was obtained from all subjects.

### Clinical study: design

After a 2-week run-in period, during which the subjects consumed their habitual normal protein diet, each subject was randomly assigned to receive either a high protein diet (HP group; i.e., target of > 25% of total energy intake derived from protein intake) or a low protein diet (LP group; i.e., target of 9%). During the first week of the run-in period, the subjects completed a 7-day dietary record, allowing us to calculate total energy intake and dietary macronutrient composition. With this information, we composed individually adapted diets, isocaloric to the habitual diet of the subjects and taking into account personal preferences. In the HP group, diet was also supplemented with 20 g protein powder per day (Resource, Nestlé Healthcare Nutrition, Switzerland), while protein was replaced by digestible carbohydrates in the LP group. Additionally, in both treatment groups, we aimed to keep fat and fiber intake as constant as possible. These individually adapted diets were presented to the subjects by giving each of them 3 lists with 7 suggestions for breakfast, lunch and dinner, respectively. Depending on their caloric needs, they were also allowed to choose two or three snacks from a 4th list. During the second week of intervention the subjects again completed a 7-day dietary record to evaluate the actual food intake. During the run-in period and at the end of the intervention period, we also collected blood after an overnight fast and urine (48h urinary collection) of each subject. All samples were stored at– 80°C until further analysis.

### Preclinical study: design

Wild-type Friend leukemia virus B (FVB) mice were bred and housed at the Central Animal Laboratory of the Radboud University Medical Center. Animals were divided in two groups of 10 animals and provided either a control diet (21% crude protein) or a high protein diet (45% crude protein; Ssniff Spezialdiäten GmbH, Soest, Germany) for 21 days. All animals had free access to food and water. After 21 days, blood was collected in lithium-heparin tubes by cardiac puncture under isoflurane anesthesia and animals were sacrificed by cervical dislocation. Plasma was immediately snap frozen in liquid nitrogen and stored at– 80°C until further analysis. All experiments were approved by the local Animal Welfare Committee of the Radboud University Medical Center (RU-DEC 2012–292), in accordance with the directive for animal experiments (2010/63/EU) of the European Parliament.

### Analytical procedures

Dietary records were analyzed using the online food calculator ‘Nubel’ and information on standardized quantification of food products. Blood and urine human samples were used to measure creatinine and urea with standard laboratory techniques. In addition, human and mice samples were analyzed for a panel of metabolites, focusing on selected tryptophan and phenolic compounds. All analyses were based on single measurements. With respect to the tryptophan metabolites, we measured tryptophan, indoxyl sulfate, indoxyl glucuronide, indole-3-acetic acid, kynurenine, kynurenic acid and quinolinic acid. Phenolic compounds included *p*-cresyl sulfate, *p*-cresyl glucuronide, phenyl sulfate, phenyl glucuronide and phenylacetic acid. All metabolites were quantified using a dedicated liquid chromatography—tandem mass spectrometry (LC-MS/MS) method. Deuterated kynurenic acid was added as an internal standard for quantification, as described previously [24]. Before chromatography an aliquot of plasma was diluted in H_2_O (1:1) and deproteinized with perchloric acid (final concentration 3.3% (v/v)). Next, samples were centrifuged at 12,000 x g for 3 min. Clear supernatant was injected into the LC-MS/MS system that consisted of an Accela HPLC system coupled to a TSQ Vantage triple quadropole mass spectrometer (Thermo Fischer Scientific, Breda, the Netherlands) equipped with a C18 HPLC column (Acquity UPLC HSS T3 1.8 μm; Waters, Milford, MA). The autosampler temperature was set at 8°C and the column temperature at 40°C. The flow rate was 350 μL/min. Eluent solvent A consisted of 10 mM NH4-acetate, solvent B was 5 mM NH4-acetate and 0.1% formic acid and solvent C was 100% methanol. Samples (10 μL) were injected three times. The first injection addressed the basic negative components, including indoxyl sulfate, *p*-cresyl sulfate, phenyl sulfate and phenylacetic acid, because these components are stable within the acidic environment for up to 16h. The second injection addressed the acidic negative components, including indoxyl glucuronide, quinolinic acid, *p*-cresyl glucuronide and phenyl glucuronide. The third injection addressed the acidic positive components tryptophan, indole-3-acetic acid, kynurenine, kynurenic acid and quinolinic acid. The elution gradient was as follows: for the first injection the gradient was 100% solvent A to 85% solvent C in 7 min, then for the second and third injection a gradient of 100% solvent B to 85% solvent C in 7 min. The effluent from the UPLC was passed directly into the electrospray ion source. Negative electrospray ionization (first two injections) achieved using a nitrogen sheath gas with ionization voltage at 2500 Volt. The positive (third injection) electrospray ionization was achieved using a nitrogen sheath gas with ionization voltage at 3500 Volt. The capillary temperature was set at 240°C. Detection of the components was based on isolation of the deprotonated (negative electrospray; [M-H]-) or protonated molecular ion (positive electrospray; [M+H]+) and subsequent MS/MS fragmentations and a selected reaction monitoring (SRM) were carried out. The UPLC-MS/MS operating conditions and SRM transitions used for parent compounds and ion products were optimized for each component and shown in Table 1.

**Table 1:** UPLC-MS/MS operating conditions.

	Ionization mode	*m/z* parent	*m/z* product 1	CE 1 (eV)	*m/z* product 2	CE 2 (eV)	S-lens RF amplitude (V)
Phenyl acetic acid	BN	135	91	8	-	-	40
Phenyl sulfate	BN	173	80	19	93	20	86
*p*-Cresyl sylfate	BN	187	80	19	107	23	81
Indoxyl sulfate	BN	212	80	23	132	23	96
Quinolinic acid	AN	166	78	16	122	11	36
Phenyl glucuronide	AN	269	93	42	113	13	95
*p*-Cresyl glucuronide	AN	283	107	37	113	14	74
Indoxyl glucuronide	AN	308	113	17	132	26	94
Quinolinic acid	AP	168	78	23	124	11	62
Indole-3-acetic acid	AP	176	77	42	130	16	65
Kynurenic acid	AP	190	89	39	144	39	64
d5-Kynurenic acid	AP	195	121	33	149	21	102
Tryptophan	AP	205	146	18	188	10	54
Kynurenine	AP	209	146	20	192	9	56

Abbreviations: UPLC-MS/MS, ultra-performance liquid chromatography—tandem mass spectrometry; m/z, mass-to-charge ratio; CE, collision energy; RF, radiofrequency; BN, basic negative; AN, acidic negative; AP, acidic positive

With the 48h urinary collection of human subjects, we calculated the average daily urinary excretion of each metabolite, giving an estimate of its daily generation. Urinary collections were considered complete when urinary excretion of creatinine was within 2 standard deviations of the mean creatinine excretion for the geographical region of this study, derived from the INTERSALT study [25].

### Statistical analysis

Data are expressed as mean (standard deviation) for normally distributed variables or median (interquartile range: IQR) for non-normally distributed variables. Differences in demographic and dietary variables according to treatment group (HP vs. LP group) were tested using Student’s t-test, Wilcoxon rank-sum test or chi-squared test as appropriate. Changes in human solute levels (post-intervention minus baseline) were correlated with changes in dietary protein intake with Spearman's rank correlation coefficient, as well as compared with respect to treatment group using Wilcoxon rank-sum test. Differences in mice solute levels (post-intervention) between treatment groups (HP vs. control diet group) were also tested with Wilcoxon rank-sum test. For all statistical analysis, P-values less than 0.05 were considered significant. All statistical analyses were performed using SAS (version 9.3, the SAS institute, Cary, NC, USA).

## Results

### Study population: Demographic and dietary characteristics

**Fig 1:**
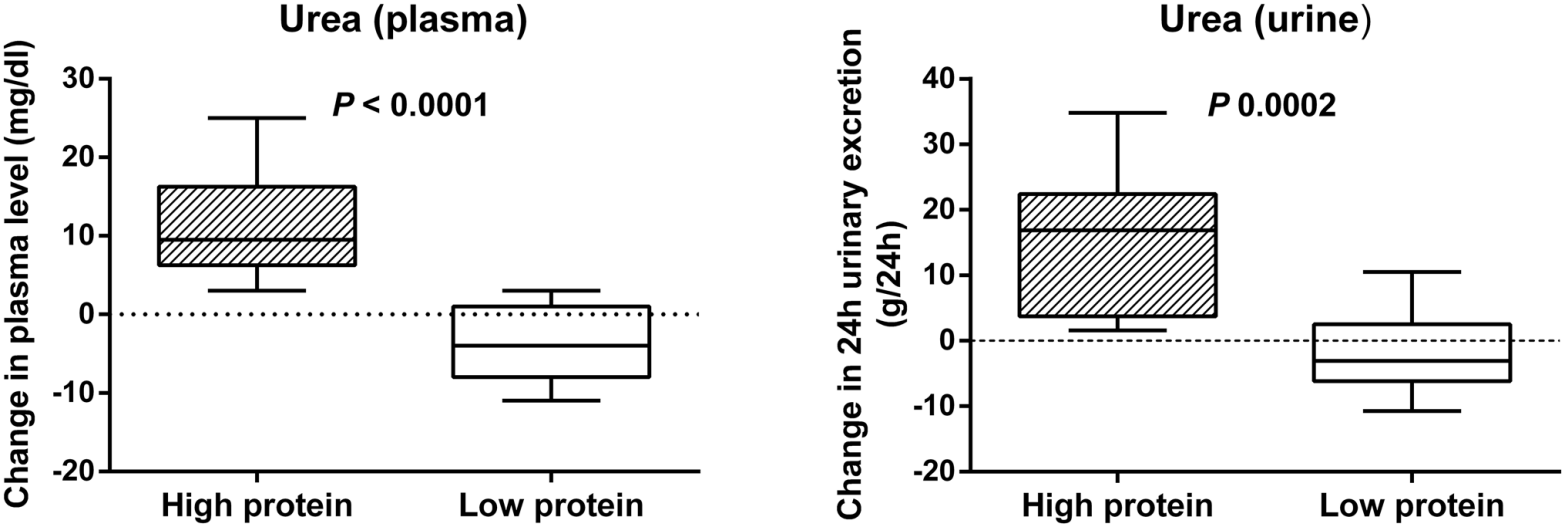
Protein intake and urea. Influence of a high vs. low protein diet on change in plasma urea and 24h urinary excretion of urea.

A total of 29 healthy subjects were included for analysis. Of these, 14 subjects received a high protein diet and 15 subjects were allocated to a low protein diet (Table 2). There were no significant differences in age, gender and body mass index between both groups. During the run-in period, we observed no differences in both total energy intake and macronutrient intake (i.e., protein, carbohydrate, fiber and fat intake). On the other hand, as intended, protein intake was significantly higher during intervention in the HP group (median of 124.6 g/day (122.7–167.0) or 1.9 g/kg/day (1.9–2.3)) than in the LP group (50.0 g/day (43.3–58.5) or 0.8 g/kg/day (0.7–1.0)) (*P* < 0.0001). Relative energy intake from dietary protein amounted to a median of 27% (IQR 25–29) in the HP group vs. 13% (IQR 12–13) in the LP group. Plasma urea and 24h urinary excretion of urea also significantly increased in the HP vs. LP group (*P* < 0.0001 and *P* 0.0002, respectively; Fig 1). Total energy intake, fiber intake and fat intake were not statistically different between both groups.

**Table 2:** Study population.

Variable	Overall (n = 29)	High protein (n = 14)	Low protein (n = 15)	*P*
Age (y)	22 (20–23)	22 (21–23)	21 (19–24)	0.58
Gender: male/female (%)	9/20 (31.0/69.0)	3/11 (21.4/78.6)	6/9 (40.0/60.0)	0.43
Body mass index (kg/m^2^)	21.03 (19.61–22.91)	22.56 (20.55–23.18)	20.98 (19.57–22.23)	0.23
*Run-in diet*:				
Calories (Kcal/day)	2053.8 (379.4)	2023.1 (322.0)	2082.3 (435.7)	0.68
Protein (g/day)	74.1 (67.5–84.3)	73.0 (67.0–78.1)	74.1 (67.5–95.0)	0.59
Carbohydrate (g/day)	246.0 (67.0)	235.8 (56.5)	255.5 (76.3)	0.44
Fiber (g/day)	17.4 (5.5)	15.9 (3.5)	18.7 (6.7)	0.18
Fat (g/day)	94.3 (35.5)	83.4 (37.6)	104.4 (31.3)	0.11
*Intervention diet*:				
Calories (Kcal/day)	1875.3 (383.4)	2000.6 (293.0)	1758.3 (428.8)	0.09
Protein (g/day)	84.3 (50.0–124.5)	124.6 (122.7–167.0)	50.0 (43.3–58.5)	< 0.0001
Carbohydrate (g/day)	230.3 (63.4)	204.0 (40.1)	254.9 (72.2)	0.03
Fiber (g/day)	17.1 (6.3)	15.1 (3.5)	19.0 (7.7)	0.09
Fat (g/day)	93.1 (30.7)	87.2 (25.8)	98.7 (34.6)	0.3

### Tryptophan metabolites

We investigated the influence of dietary protein intake on the tryptophan metabolites. When correlating changes in protein intake during intervention with changes in plasma level and 24h urinary excretion of the various metabolites, we noted significant correlations with 24h urinary excretion of tryptophan (ρ 0.47, *P* 0.01), plasma indoxyl sulfate (ρ 0.56, *P* 0.002), 24h urinary excretion of indoxyl sulfate (ρ 0.70, *P* < 0.0001), 24h urinary excretion of indoxyl glucuronide (ρ 0.44, *P* 0.02), plasma kynurenic acid (ρ 0.42, *P* 0.02), 24h urinary excretion of kynurenic acid (ρ 0.61, *P* 0.0008) and 24h urinary excretion of quinolinic acid (ρ 0.47, *P* 0.01). There was no significant relationship with plasma tryptophan (*P* 0.18), plasma and 24h urinary excretion of indole-3-acetic acid (*P* 0.63 and *P* 0.27, respectively), and plasma and 24h urinary excretion of kynurenine (*P* 0.74 and *P* 0.17, respectively). Measurements of plasma indoxyl glucuronide and plasma quinolinic acid were below their limits of detection.

**Fig 2:**
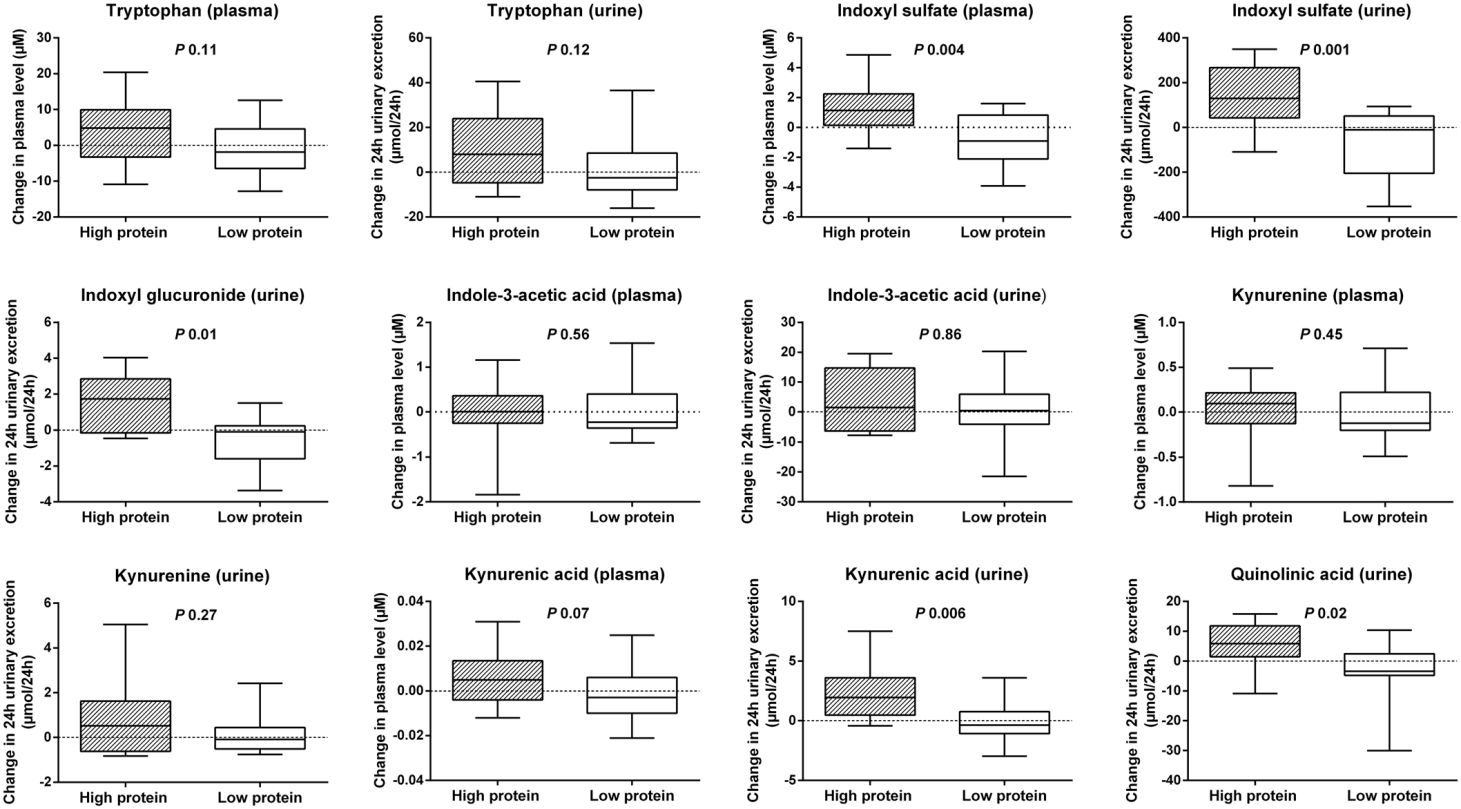
Protein intake and tryptophan metabolites. Influence of a high vs. low protein diet on change in plasma level and 24h urinary excretion of tryptophan metabolites.

Next, we compared changes in plasma level and 24h urinary excretion of the various tryptophan metabolites in the HP vs. LP group (Fig 2), observing a significant increase in plasma level and 24h urinary excretion of indoxyl sulfate (*P* 0.004 and *P* 0.001, respectively), 24h urinary excretion of indoxyl glucuronide (*P* 0.01), 24h urinary excretion of kynurenic acid (*P* 0.006) and 24h urinary excretion of quinolinic acid (*P* 0.02).

### Phenolic metabolites

The influence of dietary protein intake was also explored with respect to phenolic metabolites. Changes in protein intake were correlated with changes in plasma level and 24h urinary excretion of these compounds, only observing a significant relationship with 24h urinary excretion of *p*-cresyl sulfate (ρ 0.40, *P* 0.04). There was no significant correlation with plasma *p*-cresyl sulfate (*P* 0.28), plasma and 24h urinary excretion of *p*-cresyl glucuronide (*P* 0.49 and *P* 0.22, respectively), plasma and 24h urinary excretion of phenyl sulfate (*P* 0.25 and *P* 0.26, respectively), and 24h urinary excretion of phenyl glucuronide (*P* 0.37). Measurements of plasma and urine phenylacetic acid, and plasma phenyl glucuronide were below their limits of detection.

**Fig 3:**
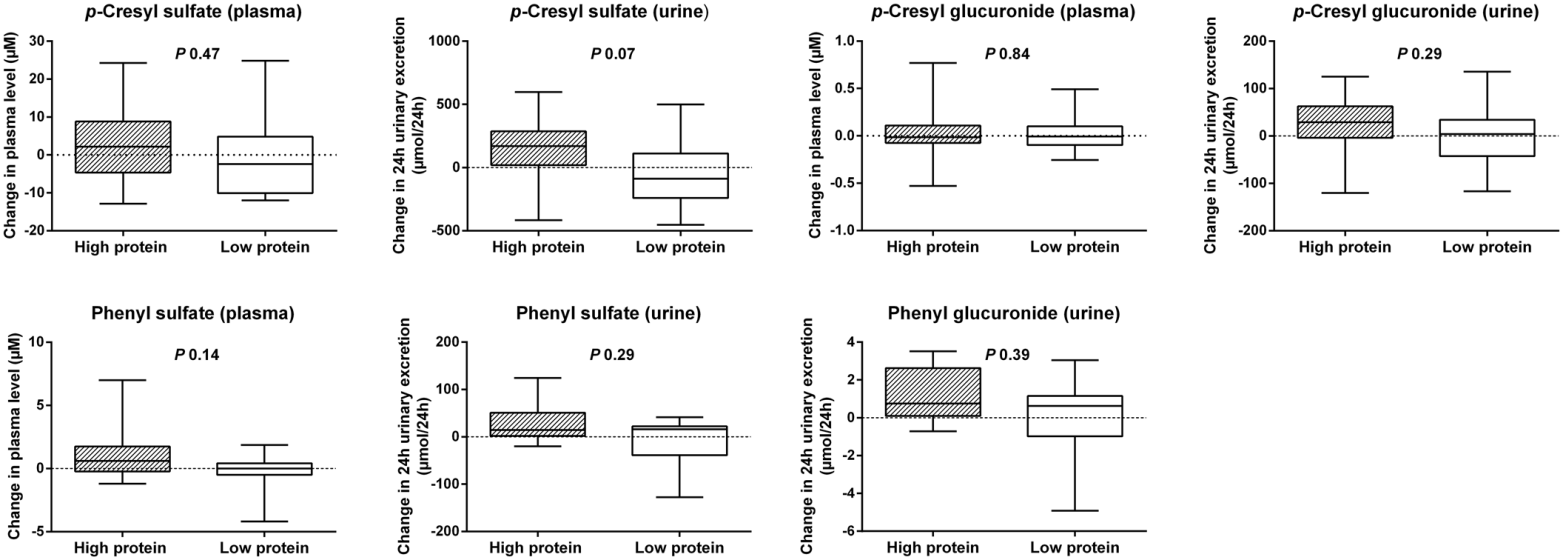
Protein intake and phenolic metabolites. Influence of a high vs. low protein diet on change in plasma level and 24h urinary excretion of phenolic metabolites.

In addition, changes in plasma level and 24h urinary excretion were compared in the HP vs. LP group (Fig 3). We noted an increasing, though not formally significant, trend in 24h urinary excretion of *p*-cresyl sulfate (*P* 0.07). However, there were no between-group differences with respect to the other metabolites.

### Preclinical study

**Fig 4:**
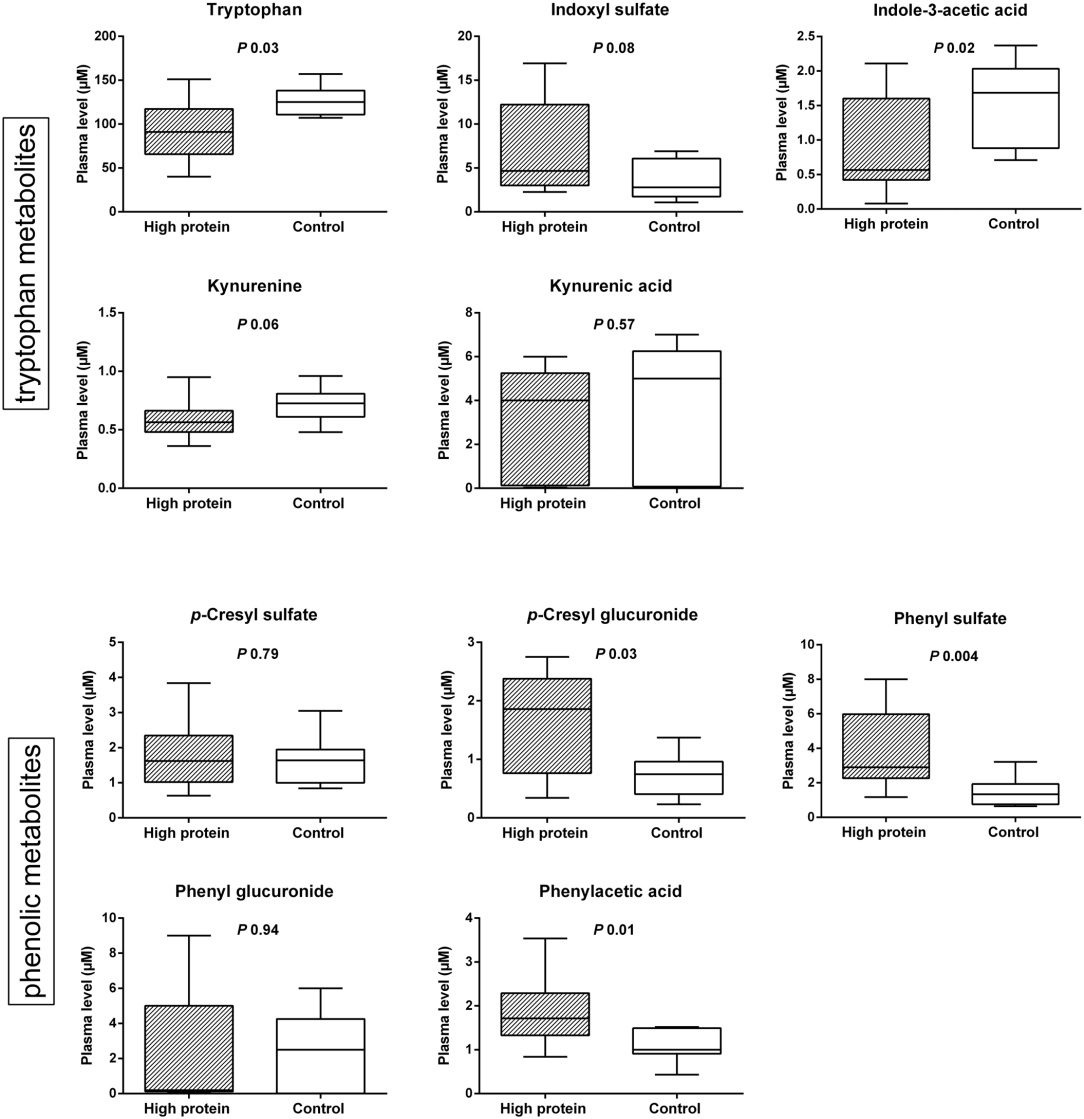
Protein intake and metabolites in mice. Influence of high protein vs. control diet on plasma level of tryptophan and phenolic metabolites in mice.

Mice were subjected to either a high protein or a control diet with measurement of plasma samples after intervention (Fig 4). At baseline and after intervention, there were no differences in body weight between both groups (*P* 0.27 and *P* 0.38, respectively). With respect to the tryptophan metabolites, we observed significantly lower levels of plasma tryptophan (*P* 0.03) and indole-3-acetic acid (*P* 0.02) in the HP group with also a trend of higher levels of plasma indoxyl sulfate (*P* 0.08) and lower levels of plasma kynurenine (*P* 0.06). Measurements of plasma quinolinic acid and indoxyl glucuronide were below their limits of detection. Furthermore, with regard to the phenolic metabolites, we observed significantly higher levels of plasma *p*-cresyl glucuronide (*P* 0.03), phenyl sulfate (*P* 0.004) and phenylacetic acid (*P* 0.01) in the HP group.

## Discussion

In this study, we investigated the influence of dietary protein intake on the mammalian metabolome, focusing on selected tryptophan and phenolic metabolites. The key findings are: (i) protein intake influences mammalian plasma levels and generation of various metabolites; (ii) protein intake interferes with both the endogenous and colonic microbial metabolism; (iii) substantial differences are observed between human and mouse metabolic response to protein intake.

Although increasingly used in the treatment of obesity, the long term health effects of high protein diets are still unclear [2, 3]. Additionally, there has been an historical interest in low protein diets in the treatment of patients with CKD [5]. However, the influence of dietary protein intake on the mammalian metabolome is not well understood, although it can be hypothesized that potential health effects are also mediated by changes in specific metabolites.

Therefore, we investigated the influence of dietary protein intake on the mammalian metabolome by allocating healthy subjects and mice to a high protein or a low protein (human)/control (mice) diet, focusing on selected tryptophan and phenolic compounds. In human subjects, we observed significant changes in plasma levels and generation of indoxyl sulfate, indoxyl glucuronide, kynurenic acid, quinolinic acid, and to a lesser extent, *p*-cresyl sulfate (all higher in HP group), pointing to an impact of protein intake on solutes derived from both endogenous and colonic microbial metabolism. Regarding the endogenous metabolites, higher levels of kynurenic acid have already been associated with inflammation and cardiovascular disease in the general population, as well as in patients with renal dysfunction [10, 26]. In addition, kynurenic acid and quinolinic acid have also been related with development of CKD [13]. The relevance of the colonic microbial metabolites have been mainly demonstrated in patients with pre-existing renal disease [14–18, 20], although recent findings also suggest the importance of these solutes independent of or preceding renal dysfunction [27, 28].

The significant increase in plasma levels and generation of indoxyl sulfate in the high vs. low protein diet group are in agreement with a study of Patel *et al*., demonstrating higher urinary excretion of indoxyl sulfate in omnivores vs. vegetarians with higher protein intake observed in omnivores [29]. On the other hand, we could only note a trend of higher levels of *p*-cresyl sulfate in the high vs. low protein diet group, while there was also a substantially higher generation of *p*-cresyl sulfate in the group of omnivores in the abovementioned study. This apparent discrepancy may be explained by the concomitant lower intake of vegetables in the omnivores, while fiber intake was kept constant in the current study. Interestingly, a recent study exploring the effects of prebiotics on serum levels of *p*-cresyl sulfate and indoxyl sulfate in hemodialysis patients could also demonstrate a decreasing effect on serum *p*-cresyl sulfate, but not on indoxyl sulfate [30]. This may suggest that absolute and relative differences in protein and fiber intake, besides other, yet unknown, dietary or microbial factors are responsible for differential changes in generation of these two colonic microbial metabolites.

Concomitantly, we explored the influence of dietary protein intake in mice, observing significant changes in plasma levels of tryptophan, indole-3-acetic acid (all lower in HP group), *p*-cresyl glucuronide, phenyl sulfate and phenylacetic acid (all higher in HP group), as well as a clear trend of higher levels of plasma indoxyl sulfate and lower levels of plasma kynurenine. As metabolite changes were markedly different between human subjects and mice, these findings suggest inter-species differences in not only the endogenous metabolism, but also in the colonic microbial metabolism. Regarding the endogenous metabolites, although there is substantial genetic homology between mice and human, there are still notable differences in gene expression, also causing important differences in the endogenous metabolism of xenobiotics [31] and possibly explaining the dissimilar effect of protein intake on the endogenous metabolites measured in the current study. Furthermore, while it is assumed that mouse and human models share a common core gut microbial composition with respect to the phylum and genus levels, there are still substantial dissimilarities in the bacterial relative abundance [32], which, besides environmental factors, could explain inter-species differences in the fecal metabolome [33], and therefore also the differential impact of dietary protein intake on plasma levels of the microbial metabolites in this study. Although this may question the feasibility of translating mouse to human models of colonic protein fermentation, especially with respect to protein intake, potential human-mouse dissimilarities due to differences in type and/or amount of ingested protein (e.g., 45% relative protein intake in HP mice group vs. target of 25% in HP human group) have to be excluded.

As this study only included healthy subjects and animals, the influence of dietary protein intake on the metabolome in patients with CKD remains to be elucidated. While a recent study has suggested that a protein diet of 0.3 g/kg/day reduced indoxyl sulfate levels in patients with CKD stage 3 [34], the impact on other metabolites requires further study. In addition, whether a more modest reduction in protein intake to 0.8 g/kg/day, as generally recommended in order to offset the risk of malnutrition [35], has the same effect on these metabolites is unknown. It must also be noted that the protein assimilation process in the small intestine is impaired due to renal failure, causing an increased colonic availability of protein, thereby promoting protein fermentation [36]. Although it can, therefore, be hypothesized that similar changes in dietary protein intake have a more pronounced effect on colonic microbial metabolism in CKD patients, this needs to be proven.

There are limitations to our study. First, we investigated the influence of dietary protein intake on the mammalian metabolome by focusing on selected tryptophan and phenolic compounds. As samples were not analyzed by an untargeted metabolomic approach, the effect of protein intake on other metabolites could not be derived. Second, our study population solely consisted of healthy subjects of Caucasian origin. Care must be taken to extrapolate our data to other populations. Finally, as there were no measurements of urine samples in mice, we could only investigate the influence of protein intake on their plasma levels, not on urinary excretion rates. Still, we do not expect that this would have altered our main findings.

## Conclusions

We demonstrated that dietary protein intake affects the mammalian metabolome by interference with both the endogenous and colonic microbial metabolism. Metabolite changes are dissimilar between human subjects and mice, pointing to inter-species metabolic differences with respect to protein intake. The relevance of these findings to the general population as well as patients with CKD needs further investigation.
